# Risk Factors and Spatial Distribution of Gastrointestinal Parasites in Backyard Poultry Production Systems in Central Chile

**DOI:** 10.3390/vetsci12050448

**Published:** 2025-05-07

**Authors:** Bruno Cantin-Rosas, Mariela Luján Tomazic, Anabel Elisa Rodríguez, Nikita Enciso, Juliette Brante-Bernier, Patricia Honores, Catalina Godoy-Alfaro, Claudio Abarca, Raúl Alegría-Morán, Galia Ramirez-Toloza

**Affiliations:** 1 Laboratorio de Parasitología y Enfermedades Parasitarias, Departamento de Medicina Preventiva Animal, Facultad de Ciencias Veterinarias y Pecuarias, Universidad de Chile, Santa Rosa 11735, La Pintana, Santiago 8820808, Chile; brunoignaciocr@gmail.com (B.C.-R.); nikita.enciso@ug.uchile.cl (N.E.); juliette.brante@ug.uchile.cl (J.B.-B.); patyhonoresperez@gmail.com (P.H.); cata.godoy.a@gmail.com (C.G.-A.); claudio.abarca@ug.uchile.cl (C.A.); 2 Instituto de Patobiología Veterinaria (IPVET), Instituto Nacional de Tecnología Agropecuaria (INTA), Consejo Nacional de Investigaciones Científicas y Técnicas (CONICET), de Los Reseros y Nicolás Repetto s/n, Hurlingham, Buenos Aires 1686, Argentina; rodriguez.anabel@inta.gob.ar; 3 Facultad de Farmacia y Bioquímica, Universidad de Buenos Aires, Av. Junín 954, Ciudad Autónoma de Buenos Aires C1113, Argentina; 4 Programa de Doctorado en Ciencias Silvoagropecuarias y Veterinarias, Universidad de Chile, Santa Rosa 11315, La Pintana, Santiago 8820808, Chile; 5 Escuela de Medicina Veterinaria, Sede Santiago, Facultad de Recursos Naturales y Medicina Veterinaria, Universidad Santo Tomás, Ejercito Libertador 146, Santiago 8370003, Chile; ralegria2@santotomas.cl

**Keywords:** backyard poultry production system, risk factors, *Eimeria* spp., *Capillaria* spp., *Ascaridia galli*, *Trichostrongylus* spp., *Heterakis gallinarum*

## Abstract

Gastrointestinal parasites impact the health and productivity of domestic birds and may even be more common in production systems with lower biosafety conditions. In this context, backyard poultry production systems (BPPS), defined as small-scale family production systems, could be more affected. However, information about its epidemiology is limited in the Central Zone of Chile. This study aimed to determine the risk factors and spatial distribution of gastrointestinal parasites in BPPS in Central Chile. Thus, feces samples were collected from 51 backyards and analyzed using copro–parasitological techniques. In parallel, an epidemiological survey was conducted on the farmers, and the data were analyzed by multivariable logistic regression to identify risk factors. Spatial analysis was performed with the detected parasite species to determine high-risk clusters. *Eimeria* spp. was the most frequently detected parasite (72.5%), followed by *Capillaria* spp. (50.9%) and *Ascaridia galli* (49%). Regarding parasitic burden, nearly 90% of BPPS showed low parasitic burden for *Eimeria* spp. and helminths. In turn, the availability of potable drinking water (95% CI: 0.054–0.905; *p* = 0.036) and proper ventilation of the pens (95% CI: 0.003–0.429; *p* = 0.009) reduced the presence of parasites. Spatial high-risk clusters were detected for *Eimeria* spp. (RR = 2.60; *p*-value < 0.0001), *A. galli* (RR = 2.93; *p*-value = 0.021), and *Trichostrongylus* spp. (RR = 5.85; *p*-value = 0.050).

## Simple Summary

This study evaluated the presence of gastrointestinal parasites in backyard poultry production systems (BPPS) in Central Chile, where biosecurity practices are often limited. A total of 51 backyards were assessed using fecal analysis and epidemiological surveys. The most commonly detected parasites were *Eimeria* spp. (72.5%), *Capillaria* spp. (50.9%), and *Ascaridia galli* (49%). Although the parasite burden was mainly low, protective factors such as access to clean drinking water and good ventilation were identified. High-risk geographic clusters for infection were also detected.

## 1. Introduction

Poultry production systems can be categorized into industrial (large, medium, and small producers) and small-scale backyard farms, also called backyard poultry production systems (BPPS) [1,2]. The latter are characterized by minimal biosecurity, informal flock management, and distinct epidemiological profiles [3]. From an economic perspective, BPPS provides a supplemental income, is easy to operate, and ensures the availability of eggs and meat in rural areas [2]. However, from a health perspective, BPPS may be less aware of the legislation, rules, and biosecurity practices implemented in the livestock sector [4], which could be involved in transmitting both endemic and non-endemic diseases across many countries [5]. However, the surveillance and diagnosis of other infections affecting productive parameters, such as parasitic diseases, are often restricted to post-mortem examinations or anecdotal evidence [6].

Gastrointestinal parasitoses impact health, welfare, and productivity worldwide across various agricultural systems, including the poultry industry [7]. In Chile, parasitic infections caused by protozoa and helminths have been reported in domestic chickens [8]. *Eimeria* spp. is significant among protozoan parasites as it affects the large and small intestines of hens, damaging enterocytes and the connective tissue mucosa [9]. Infection may arise from one or more species of *Eimeria* spp., which exhibit differing levels of pathogenicity and are associated with production losses [9]. Additionally, more than 30 helminth species affecting domestic chickens have been identified, including nematodes (roundworms) such as *Ascaridia galli*, *Heterakis gallinarum*, and *Capillaria* spp.; cestodes (tapeworms) like *Raillietina* spp.; and trematodes (flukes). Depending on the parasitic burden, these organisms may cause symptoms such as diarrhea, bleeding, anorexia, reduced daily weight gain, and even increased mortality rates [7].

Multiple risk factors are associated with gastrointestinal parasitism. Animal-specific factors such as age; external factors like insufficient veterinary assistance, inadequate disease control strategies, poor management, high animal density, unsanitary bedding and pens, feeding and water administration methods involving fecal–oral transmission of parasitic agents, inadequate facilities, and a lack of knowledge among producers; and environmental factors like temperature and humidity have been identified as significant risk factors [6,10,11,12]. These factors and their prevalence may vary by country, continent, and farming system. In Central Chile, previous records of the circulation of parasitic species in BPPS are either nonexistent or limited, primarily associated with cattle rather than birds due to their stronger link with zoonotic transmission events. On the other hand, considering that BPPS are not part of formal records, and only those with commercial permits are registered within the National Agricultural Census, the current Chilean BPPS population is unknown.

Conversely, infectious agents like bacteria and viruses can interact with specific gastrointestinal parasites, generating synergistic or antagonistic effects. For example, coinfection with *Salmonella* spp. and *Eimeria* spp. affects the gut microbiota, increasing liver *Salmonella* colonization and fat deposition in turkeys [13]. However, the impact of parasitic infection as a risk factor for another parasitosis has not been examined. Thus, this study aimed to determine risk factors and spatial distribution linked to gastrointestinal parasitism in domestic chickens in Central Chile.

## 2. Materials and Methods

### 2.1. Sample Size and Study Area

#### 2.1.1. Sample Size

Taking into account the previously discussed background of nonexistent or limited information on parasitoses in BPPS in Chile, BPPS being the epidemiological unit, a sampling approach for pathogen detection was determined, assuming prior freedom and unknown population size, following the equation [14]:

n = ln α/ln(1 − p),(1)

where n corresponds to the sample size, α is the type I error (confidence level), and p is the expected minimum prevalence. The confidence level was set at 95%, and an unknown population size approach was used, considering the uncertainty about the current Chilean BPPS population, which can be treated as infinite, with a minimum expected prevalence of 10% (regarded as conservative since a higher prevalence is anticipated). A sample size equivalent to 29 BPPS was established. However, this study involved collecting samples from 51 different backyards, facilitated by the collaboration of PRODESAL users (a state organization supporting family farming) across central Chile.

#### 2.1.2. Study Area

Fifty-one BPPS located in three different regions of Chile were sampled from July to August: twenty from the Metropolitan region, twenty-five from the Libertador General Bernardo (LGB) O’Higgins region, and six from the Valparaíso region.

#### 2.1.3. Sampling Method

The BPPS were chosen randomly from a registry of PRODESAL users. After signing an informed consent form, door-to-door consultations were held. A previously validated epidemiological survey was also conducted through a semi-structured face-to-face interview following the signing of the informed consent form [15]. Briefly, fresh fecal pellets were collected from the chicken coops until approximately 50 g was obtained. The collection was carried out following a “W” route through the unit, starting from one corner, with a sample collected every two to five steps.

A GPS device recorded the exact geolocation of the BPPS. Samples were stored in large, airtight plastic bags, then moved to and refrigerated at 4 °C in the Laboratory of Parasitology and Parasitic Diseases of the Faculty of Veterinary and Animal Sciences of the University of Chile until they were processed.

### 2.2. Parasitological Examination for Gastrointestinal Parasite Identification

#### 2.2.1. Flotation Technique

Microscopic analysis used qualitative coprological examination methods, concentrating parasitic elements (oocysts from parasitic protozoa and eggs from gastrointestinal helminths). The collected feces were homogenized, and 5 g of feces was examined using standard flotation methods [16]. A saturated sodium chloride flotation solution (1.2 Mean Specific Gravity) was prepared. Briefly, each sample was sieved into a plastic tube filled with saturated sodium chloride solution and covered with a coverslip. After 10 min, the coverslip was carefully removed, placed over a glass slide, and analyzed microscopically to detect the presence of eggs or oocysts. Samples were examined under a light microscope using 10× and 40× objectives, with a calibrated ocular micrometer employed to measure the eggs’ dimensions and classify them by genus and species [17]. This method was performed in duplicate for each sample.

#### 2.2.2. McMaster Counting Technique

Samples where parasitic forms were identified were further subjected to the McMaster counting technique to estimate the parasitic burden (intensity of infection). Five grams of feces were dissolved in 75 mL of saturated sodium chloride solution. Both chambers of the McMaster plate (0.15 mL each) were filled with the solution. The oocysts or eggs were counted in each chamber, and the total number was multiplied by 100 and then divided by 2 to express the oocysts per gram of feces (OPG) or eggs per gram of feces (EPG) for each sample and genus detected [18].

The parasitic burden was calculated according to the literature. For *Eimeria* spp., the burden was classified as low (<1800 OPG), medium (1800–6000 OPG), and high (>6000 OPG) [19]. For nematodes, the count was classified as low (<500 EPG), medium (500–2000 EPG), and high (>2000 EPG) considering artificial infections performed with *Capillaria* spp. [20], *Heterakis* sp. [21], *A. galli* [22], and Trichostrongylus [23].

### 2.3. Data Management and Risk Factors and Spatial Analysis

#### 2.3.1. Questionnaire Survey

A survey was conducted with each farm manager or foreman to identify risk factors for parasitic infections. The main topics included in the questionnaire to determine risk factors for parasitosis are summarized in Table 1 (the complete survey is in Supplementary Material S1).

The instrument was validated on family farmers/BPPS in Argentina [15] and re-validated in a pilot with PRODESAL users in the LGB O’Higgins region.

#### 2.3.2. Multivariable Regression Analysis

Considering the binary/dichotomous nature of the response variable, with Y having only two possible values, 0 and 1 (Y = 0 or Y = 1), multivariable logistic regression was used to determine the factors that modify the risk of the presence of gastrointestinal parasites. 

First, a univariable logistic regression analysis was performed with all the variables considered in this study, where only those variables with a *p*-value < 0.15 were chosen (liberal p criterion) for the multivariable model building. Subsequently, the selected variables were evaluated using the Spearman correlation test (quantitative variables) and the Fisher exact test or Chi-square (qualitative variables) to evaluate collinearity and association between dependent variables. Biological and epidemiological coherent interactions were assessed. Goodness-of-fit was evaluated with the Hosmer–Lemeshow test. All data analyses were performed with R statistical software version 4.2.2 [24] and RStudio version 2022.12.0+353 [25].

#### 2.3.3. Spatial Analysis

Spatial scan statistic was applied to the presence of parasite species using the Kulldorff space exploration statistic to identify spatial clusters [26]. Considering the binary nature of the data (presence = 1 or absence = 0), the Bernoulli model was used to assess local clusters [27]. All the analyses were performed using SatScan software version 9.4.2 [28], all the parameters were fixed by default, and only high-risk clusters were searched. Maps were generated using QGis 3.6 Noosa [29].

## 3. Results

### 3.1. Frequency of Gastrointestinal Parasites in BPPS in Central Chile

**Figure 1:**
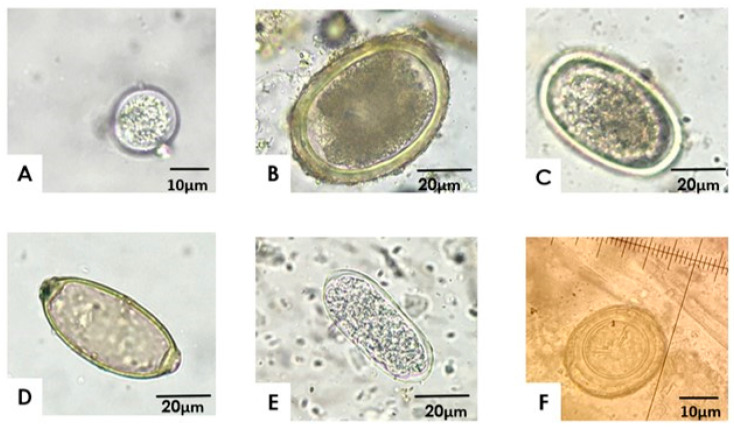
Gastrointestinal parasites identified in the backyard poultry production systems in Central Chile. Samples were analyzed using flotation and examined under a light microscope at 400× magnification. The images represent oocysts (**A**) or eggs (**C**,**D**) from the following parasites: (**A**) *Eimeria* spp., (**B**) *Ascaridia galli*, (**C**) *Heterakis gallinarum*, (**D**) *Capillaria* spp., (**E**) *Trichostrongylus* spp., (**F**) Cestode-like egg.

In 49 of the 51 BPPS (96%), at least one genus of parasites was found. The five parasitic genera and species detected include *Eimeria* spp., *A. galli*, *H. gallinarum*, *Capillaria* spp., and *Trichostrongylus*, as well as a cestode-like egg (Figure 1).

The frequency of each parasitic genus or species concerning the total samples was estimated. *Eimeria* spp. had the highest frequency (72.5%), followed by *Capillaria* spp. (50.9%), *A. galli* (49%), *H. gallinarum* (25.4%), *Trichostrongylus* spp. (25.4%), and cestode-like eggs (2%) (Table 2). Considering the 49 samples with at least one parasite, in 14 samples, only one parasite was found (28.6%), and in 35 samples, multiple genera were present (71.4%). The most frequent co-occurrence corresponded to *Eimeria* spp. and *Capillaria* spp. (*n* = 6), followed by *Eimeria* spp. and *A. galli* (*n* = 4), and then by *Eimeria* spp., *Capillaria* spp., and *A. galli* (*n* = 2).

### 3.2. Estimated Gastrointestinal Parasite Burden in BPPS in Central Chile

The estimated burden of gastrointestinal parasites was measured for all the identified parasites. For *Eimeria* spp., the burden was low in 88.2% of BPPS, medium in 7.84%, and high in only 3.9%. For nematodes, 94–100% of BPPS presented a low estimated burden (Table 3).

The descriptive statistics show that in the case of *Eimeria* spp. for a low estimated burden, the mean OPG was 381.5 (standard error = 74.4; standard deviation = 452.7); for a medium estimated burden, the mean OPG was 4137.5 (standard error = 723.9; standard deviation = 1447.8); and for a high estimated burden, the mean OPG was 8800 (standard error = 425.0; standard deviation = 601.0). In the case of *H. gallinarum*, for a low estimated burden, the mean EPG was 14.2 (standard error = 3.9; standard deviation = 25.8). For *A. galli*, the mean EPG was 40.6 (standard error = 12.8; standard deviation = 86.2) for the low estimated burden. For *Capillaria* spp., the mean EPG was 36.1 (standard error = 10.9; standard deviation = 73.2) for a low estimated burden. For *Trichostrongylus* spp., the mean EPG was 23.2 (standard error = 7.4; standard deviation = 48.3) for a low estimated burden, with a mean EPG of 725.0 (standard error = 100.0; standard deviation = 141.4) for the medium estimated burden.

### 3.3. Risk Factors for Gastrointestinal Parasites in BPPS in Central Chile

Risk factors were analyzed using a multivariate logistic regression, which considered environmental factors, infrastructure, biosecurity, handling characteristics, intrinsic variables (such as age, among others), and the presence of various parasites as variables.

The presence of parasitic agents is a risk factor for specific parasites. Consequently, the presence of *Capillaria* spp. increases the risk of the presence of *Eimeria* spp. by 18.4 times (95% CI: 1.065–318.329; *p* = 0.045). In turn, systems where *Eimeria* spp. was found had a 9.7 times greater risk of finding *Capillaria* spp. (95% CI: 1.029–91.993; *p* = 0.047) (Table 4).

However, other factors are also important. A ventilation system in chicken coops reduces the risk of the presence of *Trichostrongylus* spp. by 97.7% (95% CI: 0.003–0.429; *p* = 0.009). Conversely, systems that do not sell their products (eggs or birds) in open-air markets present a 93.7% lower risk of its presence (95% CI: 0.007–0.733; *p* = 0.026). In *Capillaria* spp., systems that do not restrict or fail to control access to the system had a 98.4% lower risk of parasitic presence (95% CI: 0.001–0.542; *p* = 0.018) (Table 4).

In turn, handling factors are essential for specific parasites. Thus, for *A. galli*, the model showed that systems that provide drinking water (potable water) reduce the risk by 88.9% (95% CI: 0.054–0.905; *p* = 0.036). Additionally, systems that do not sell their products on the streets have a 92.4% lower risk of parasitic presence (95% CI: 0.011–0.691; *p* = 0.021) (Table 4).

All models presented a good fit between the data and the final model (*p* > 0.05).

### 3.4. Spatial Analysis for Gastrointestinal Parasitosis in BPPS in Central Chile

**Figure 2:**
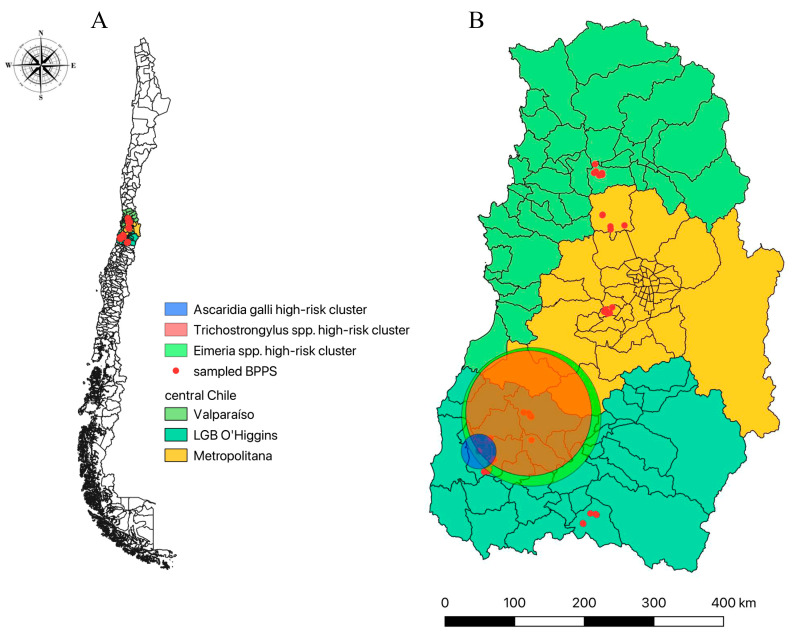
Spatial distribution of sampled BPPS from central Chile, showing (**A**) a general view of the study area; (**B**) the studied regions, showing the distribution of sampled BPPS within the three studied regions and spatial location of statistically significant high-risk clusters for *Eimeria* spp. presence (green circle), *Trichostrongylus* spp. presence (clear red circle), and *Ascaridia galli* presence (blue circle).

Statistically significant high-risk spatial clusters were detected for two out of five of the detected parasite species, showing that the *Eimeria* spp. high-risk cluster (RR = 2.60; *p*-value < 0.0001) and *A. galli* high-risk cluster (RR = 2.33; *p*-value = 0.021) are located in the LGB O’Higgins region (Figure 2). A borderline statistically significant (*p* = 0.05) high-risk (RR = 5.85) cluster was detected for *Trichostrongylus* spp. (Table 5). This cluster is also in the LGB O’Higgins region (Figure 2).

## 4. Discussion

Poultry production in Chile focuses on two primary areas: meat and egg production. The industry is now highly technological and production-intensive, meeting domestic and export market demands. However, it is diverse and spans the entire country. It comprises large companies that supply most domestic markets, commercial producers equipped with basic facilities to engage in local sales (with over 150 hens), and home or backyard production for self-consumption, primarily producing eggs [30]. However, these BPPS are typically not involved with production organizations, which may affect their knowledge and awareness of legislation and statutory requirements. Without quality assurance criteria, small-scale producers may also have less motivation to adopt effective biosecurity and management practices [4]. Consequently, these systems have shown greater susceptibility to infectious and parasitic diseases, depending on the level of biosecurity [31]. However, the topographic and climatic conditions, season, farm management, and breed of birds may also be involved [32].

In poultry, as in other production systems, gastrointestinal parasitism has an economic impact, primarily linked to poor growth, reduced egg production, fertility issues, and mortality due to acute infections [33]. In this current study, *Eimeria* spp., a parasite responsible for coccidiosis, was the most frequently detected parasite in BPPS (72.5%). This result aligns with previous findings in Chile and worldwide [15]. Coccidiosis is recognized as the most economically significant parasitic disease in poultry [34]. However, the economic impact of BPPS remains unknown. In their biological life cycle, infected chickens release unsporulated oocysts into the environment through feces, where these resistant elements sporulate based on climatic conditions (25–30 °C and 60% relative humidity) [1,35,36]. In the environment, oocysts may contaminate water and food [37]. Bloody diarrhea, secondary bacterial infections, and mortality mark clinical coccidiosis. However, in many cases, coccidiosis can be asymptomatic, presenting as weight loss and reduced egg production [1]. Any of seven *Eimeria* species may infect poultry, including *Eimeria tenella*, *Eimeria maxima*, *Eimeria acervulina*, *Eimeria necatrix*, *Eimeria brunetti*, *Eimeria mitis*, and *Eimeria praecox* [9]. These species vary in prevalence and pathogenicity, with *E. acervulina* and *E. maxima* being more prevalent and *E. tenella* being more pathogenic [9]. In Chile, previous reports indicate the presence of most of these species, with *E. maxima* being the most prevalent [38]. In parallel, samples analyzed herein were used to determine the species in Chile and Argentina through molecular biology and sequencing. This study identified the seven species in both countries, with *E. mitis* (70.3%), *E. acervulina* (62.2%), *E. tenella* (59.5%), and *E. maxima* (43.2%) being the most prevalent [15].

The most common nematodes in poultry are *A. galli*, *H. gallinarum*, and *Capillaria* spp. [7]. These parasites cause direct damage to the host by inducing the breakdown of the gastrointestinal barrier and indirectly increasing susceptibility to secondary infectious diseases [3]. Here, four species/genera of nematodes were identified: *Capillaria* spp. (50.9%), *A. galli* (49%), *H. gallinarum* (25.4%), and *Trichostrongylus* spp. (25.4%). All these species have been previously described in different locations in Chile [8,39]. However, the prevalence may vary between locations, countries, and continents. A study conducted in St. Kitts, West Indies, also found a high prevalence of *Capillaria* spp. (63%), followed by *H. gallinarum* (36%) and *A. galli* (12%) [40]. Another study conducted in the Savanna subregion, Sucre, Colombia, found a 45.6% frequency for *Capillaria* spp. [41]. In contrast, a survey conducted in Alabama, USA, detected a prevalence of 26.6% for *Capillaria* spp., followed by *A. galli* and *H. gallinarum* at 20.3% [42].

The most common cestode species are *Raillietina tetragonal*, *Raillietina echinobothrida*, and *Raillietina verticillus* [3]. In Chile, five cestodes have been described in chickens: *Choanotaenia infundibulum, Davainea proglottina, Raillietina cesticillus, Hymenolepis carioca,* and *Amoebotaenia sphenoides* [8]. In this study, only one cestode-like egg was detected. However, its morphology did not correspond to the previously described cestodes. This fact emphasizes the need to further this study and conduct a molecular description of these agents. Regarding trematodes, there are no reports of flukes affecting chickens in Chile to date [8].

Since this study used pooled samples from the environment, the estimated burden should be interpreted as an indirect approximation of the parasitic pressure within the backyard, serving as a proxy for the potential infection intensity in poultry. The parasitic burden can have productive and zootechnical consequences for poultry. In this study, most of the BPPS analyzed (88.3%) exhibited a low estimated burden of *Eimeria* spp. (<1800 OPG). Nevertheless, low parasitic burdens may promote immune competence in poultry via prolonged, low-level exposure [43,44]. Nonetheless, caution must be exercised, as oocysts are still being shed into the environment [45].

On the other hand, due to the variety of *Eimeria* species that parasitize chickens, the OPG cannot reflect the pathogenicity. Thus, the same number of oocysts from different *Eimeria* species can result in varying levels of damage [46].

A study inducing artificial infections with embryonated eggs demonstrated that up to 2000 *C. obsignata* eggs do not harm the animals [20]. For *H. gallinarum*, doses exceeding 3500 eggs induce no significant weight changes [21]. In contrast, for *A. galli*, 1000 eggs over 6 weeks induce sporadic diarrhea [22]. Surprisingly, *Trichostrongylus tenuis*, a highly pathogenic nematode, can cause death in animals with just 500 larvae [23]. In this current study, most BPPS exhibited a burden lower than 500 EPG, which is regarded as a low parasitic burden. This fact corresponds with the apparent absence of symptoms in the sampled BPPS.

Multiple risk factors can explain the differences in the prevalence of different parasitoses worldwide. Some of these are the climate or season during which the samples are collected, how they were taken, the breed, and the age of laying hens. In addition, the conditions of each backyard are determining factors since elements such as animal management, individual susceptibility, infrastructure, and the conditions in which they are found influence the persistence and spread of infectious agents [19,45,47]. In this current study, the samples were collected in pools from the environment. Therefore, individual variables were not considered.

Conversely, in poultry, various microorganisms can form relationships with other infectious agents like host microbiota, viruses, bacteria, and parasites [48]. This current study found two to four parasite co-occurrences in 71.4% of BPPS. The most frequent co-occurrences were *Eimeria* spp.–*Capillaria* spp., *Eimeria* spp.–*A. galli*, and *Eimeria* spp.–*Capillaria* spp.–*A. galli.* Other combinations of co-occurrence with four distinct parasites were identified. They all contained *Eimeria* spp. and *A. galli*, along with others, lacking specific patterns. Previous studies have noted co-occurrences and increased *Eimeria* spp. and *A. galli* prevalence in various poultry production systems [1,49]. Both parasites have a direct life cycle with free-living stages that develop into their infectious form in the environment, influenced by temperature, humidity, and oxygen levels [47,50,51]. Measures to control parasitosis, such as prophylaxis, treatment, aeration, biosecurity, and vaccination, are usually implemented only in intensive production systems [52]. Conversely, co-occurrences of *Eimeria* spp. and *Capillaria* spp. have received less attention despite the high prevalence of both genera [41,53]. The multivariate logistic regression analysis reveals an association between both parasites, with *Capillaria* spp. increasing the risk of the presence of *Eimeria* spp. by 18.4 times, and *Eimeria* spp. increasing the risk of *Capillaria* spp. by 9.7 times. Exploring their interactions could unveil whether they exhibit synergistic relationship dynamics depending on their locations within target organs and the similarities in their mechanisms of action. However, these studies should be conducted individually (e.g., collecting feces from the sewer) instead of treating BPPS as the sampling unit.

Management measures include controlling water sources and feeding them to prevent the spread of contamination among chickens [6]. The *A. galli* model determined that poultry receiving potable water have an 88.9% lower risk of gastrointestinal parasites. Feeding was not recognized as a risk factor by the model. It could be linked to the method of food administration, as 93% of BPPS reported that feed administration was manual, with birds picking up food directly from the floor, which increases the risk of food contamination with feces containing sporulated oocysts or nematode eggs.

Additionally, 84.4% of farmers indicated that they do not restrict access to the site to other people, which was expected to affect the presence of gastrointestinal parasitism significantly. Previous studies have identified the impact of this variable on hens [54]. Free access allows for unrestricted entry and exit for people who can act as vectors of contaminated feces on the land—where footbaths are also crucial—but also admits the entry of new or external animals, such as wild fauna. In contrast, the *Capillaria* spp. model identified that free access reduces the risk of presence by 98.4%. This may be due to the measurement dispersing or diluting the parasitic burden in backyards, possibly spreading it outside of these or other yards despite the presence of other vectors [47].

Significance was anticipated regarding the variables of cleaning frequency and disinfectant use, given the large percentages of respondents who reported not cleaning (55.5%) and not using disinfectants (68.8%). Cleaning is vital for controlling and preventing parasitic infections. Regular cleaning diminishes the presence of potential feces, garbage, food, or contaminated water, thereby reducing the parasite burden. Additionally, using disinfectants serves as an effective hygiene method [6,11,45,54].

Another aspect closely related to infrastructure management is highlighted in the *Eimeria* spp. model, where the adequate ventilation of living areas or chicken pens reduces the risk by 97.7%. This reinforces the management guidelines that enhance airflow and reduce humidity, as these conditions (reduced airflow and high humidity) promote oocyst sporulation, the infective stage of coccidia [45].

The spatial high-risk clusters detected in this study correspond to the first evidence of parasite spatial aggregation in BPPS in Chile and part of the scarce knowledge from backyards worldwide. More global studies have identified a higher prevalence of coccidiosis and ascaridiosis in backyard chickens in tropical countries, using a higher level of spatial aggregation [1]; in the case of *Eimeria* spp., Mexico generates a spatial distribution database but the reports are related explicitly to ruminants [55].

*Ascaridia galli* and *Eimeria* spp. have been previously reported together due to both free-living stages developing into infective parasites in the environment under similar conditions [55,56,57]. This could express that the LGB O’Higgins region has the environmental conditions for developing both parasites, which results in overlapping the high-risk clusters observed in this study. Knowing the spatial distribution of the BPPS parasite population could impact in two ways. First, this could indicate environmental contamination and its impact on domestic and wild animal populations. On the other hand, it could help establish public health interventions focused on controlling or preventing those parasite species.

This study has some limitations. First, the samples were collected from July to August, characterized by higher humidity and rainfall, which favors damp conditions and less ventilation, thereby promoting the development of nematode eggs and the sporulation of coccidia [11,45]. Future studies should take into account seasonality. Second, the samples correspond to a pool collected from the environment without considering variables specific to the individual. Fecal samples were collected as pooled material from the chicken coops—enclosed, roofed resting areas where birds remain overnight—using only freshly emitted feces to ensure their origin from domestic poultry. However, because the samples represent pooled feces at the farm level, the results reflect the parasitic status of the BPPS as a whole rather than individual birds. Therefore, detecting multiple parasitic elements in a given sample may indicate *co-occurrence* within the flock but does not confirm *co-infection* at the individual level. Additionally, although no parasite genera atypical of poultry were observed, we cannot entirely rule out the possibility of minor environmental contamination inherent to extensive systems. These factors should be considered when interpreting the patterns observed in this study. Third, this study examined only two parasitic techniques, the flotation exam and the McMaster technique, which may underestimate the prevalence of other parasites in poultry systems. Different methods, such as sedimentation or specific staining like Ziehl–Neelsen, could identify other important parasites, such as cestode eggs and protozoa like *Cryptosporidium* spp., with higher sensitivity. Fourth, few recent studies in Chile explore gastrointestinal parasites in birds, and none explore the search for new genera and species incorporating molecular biology techniques. Thus, the current research identifies Strongylidae-like eggs, which probably correspond to *Trichostrongylus*, the only species recorded in Chile chickens [39].

The BPPS in Chile illustrates the vulnerability of small-scale farming families. Other studies have shown that human and animal populations in BPPS are neglected [58]. In this study, 88% of the producers indicated that they were unaware of coccidiosis as a disease, which may also suggest the risk of animals being exposed to other parasitic diseases.

Producers should implement more accessible control measures to manage or prevent acute and severe infections, integrating these strategies to minimize health concerns. This method involves monitoring animal and facility conditions, employing disinfectants like acetic acid to hinder coccidia sporulation, routinely cleaning to remove intermediate hosts such as earthworms associated with nematodes, and ensuring proper disposal of feces, waste, and contaminated feed; these practices can help decrease the parasitic burden present in these backyard environments [23,59]. This is particularly important in the case of *Eimeria* spp., which can lead to potential outbreaks in susceptible laying hens, negatively affecting productive parameters and health while increasing the risk of other diseases such as necrotic enteritis or zoonotic diseases like salmonellosis and colibacillosis [60,61].

However, it is vital to emphasize that government institutions’ advice to small producers should involve disseminating and applying knowledge regarding infrastructure management, animal care, disease control and prevention, and biosecurity measures.

## 5. Conclusions

In the BPPS of Central Chile, gastrointestinal parasites circulate, with the most prominent genus/species being *Capillaria* spp., *Eimeria* spp., and *A. galli*, although at low intensities. Additionally, these parasites are involved in parasitic associations. Significant risk factors in this study indicated that variables such as ventilation, sources of drinking water, and restricted access to farms modify the risk of gastrointestinal parasites and cause statistically significant high-risk spatial clusters for *Eimeria* spp. and *A. galli* in the LGB O’Higgins region, highlighting both the importance of proper management in BPPS and environmental surveillance. Therefore, this study suggests that epidemiological monitoring in BPPS, training producers, and distributing information on managing animals may help control these parasites and prevent future sanitation issues.

## Figures and Tables

**Table 1:** The main topics included in the questionnaire to determine risk factors for parasitic agents in backyard poultry production systems in Central Chile and the number of associated questions.

Topics	Number of Questions
Environmental conditions and management systems in animal production	13
Knowledge about coccidial disease	8
Economic and financial aspects of the operation	5
Characteristics of the chicken and production model	5
Sanitary management and animal welfare in poultry production	5
Biosecurity and cleaning protocols in animal production	4
Responsibility and management of farm personnel	4
Animal welfare and health monitoring	4
Environmental characteristics and property access	3
Basic services	3
Government support	2

**Table 2:** Number of backyards with gastrointestinal parasites identified in 51 backyard poultry production systems in Central Chile.

Parasite	Frequency of Findings	Percentage (%)	95% CI
*Eimeria* spp.	37	72.5	58.0–83.7
*Heterakis gallinarum*	13	25.4	14.8–39.9
*Ascaridia galli*	25	49.0	35.0–63.2
*Capillaria* spp.	26	50.9	36.8–65.0
*Trichostrongylus* spp.	13	25.4	14.8–39.9
Cestode-like egg	1	1.96	0.1–11.8

**Table 3:** The estimated burden of *Eimeria* and helminths in 51 backyard poultry production systems in Central Chile.

Parasite	Intensity		
	Low	Medium	High
For coccidian (OPG *)	**(<1800)**	**(1800–6000)**	**(>6000)**
*Eimeria* spp.	88.3% (45/51)	7.8% (4/51)	3.9% (2/51)
**For nematodes (EPG ^+^)**	**(<500 EPG ^+^)**	**(500–2000)**	**(>2000)**
*Heterakis gallinarum*	98.0% (50/51)	2.0% (1/51)	0.0% (0/51)
*Ascaridia galli*	100.0% (51/51)	0.0% (0/51)	0.0% (0/51)
*Capillaria* spp.	100.0% (51/51)	0.0% (0/51)	0.0% (0/51)
*Trichostrongylus* spp.	94.1% (48/51)	3.9% (2/51)	2.0% (1/51)

* OPG: oocysts per gram; ^+^ EPG: eggs per gram.

**Table 4:** Multivariate logistic regression model for determining risk factors for gastrointestinal parasites in backyard poultry production systems in Central Chile.

Model	Variables	Category	*p*-Value	OR	CI 95%	
					Lower	Superior
*Eimeria* spp.	(Intercept)		0.343	0.195	0.007	5.712
	Presence of *Capillaria* spp.	no	reference			
		yes	0.045 *	18.415	1.065	318.329
	Access restriction	control	reference			
		no control	0.097	15.24	0.61	381.023
	Ventilation system	no	reference			
		yes	0.009 *	0.033	0.003	0.429
*Trichostrongylus* spp.	(Intercept)		0.88	1.155	0.179	7.445
	Market products at fairs	sometimes	reference			
		frequently	0.599	0.381	0.01	13.934
		no	0.026 *	0.073	0.007	0.733
	Producer applies treatments	no	reference			
		yes	0.066	5.165	0.898	29.712
*Capillaria* spp.	(Intercept)		0.347	2.931	0.312	27.5
	Presence of *Eimeria* spp.	no	reference			
		yes	0.047 *	9.727	1.029	91.993
	Access restriction	control	reference			
		no control	0.018 *	0.026	0.001	0.542
	Medicines	no	reference			
		yes	0.097	3.21	0.809	12.741
*Ascaridia galli*	(Intercept)		0.01	22.702	2.137	241.137
	Water	no potable	reference			
		potable	0.036 *	0.221	0.054	0.905
	Street sale of products	frequently	reference			
		no	0.021 *	0.086	0.011	0.691
	Adult person handles the birds	no	reference			
		yes	0.069	0.18	0.028	0.988

OR: Odds ratio; CI: confidence interval. * Statistically significant.

**Table 5:** High-risk cluster for gastrointestinal parasites in backyard poultry production systems from Central Chile.

Parasite Specie	Cluster nº	Coordinates		Radius (km)	Relative Risk (RR)	*p*-Value
		Latitude	Longitude			
*Eimeria* spp.	1	−34.177200	−71.398790	41.29	2.60	<0.0001 *
*Heterakis gallinarum*	1	−33.612541	−70.929228	3.48	4.29	0.269
	2	−34.301510	−71.398110	13.81	5.44	0.828
*Trichostrongylus* spp.	1	−34.158290	−71.416580	37.3	5.85	0.050
*Capillaria* spp.	1	−33.146020	−70.797880	127.21	1.95	0.940
*Ascaridia galli*	1	−34.362870	−71.738540	10.33	2.93	0.021 *
	2	−33.151530	−70.888650	8.27	2.53	0.912

* Statistically significant.

## Data Availability

The data presented in this study are available on request from the corresponding author.

## Supplementary Materials

The following supporting information can be downloaded at: https://www.mdpi.com/article/10.3390/vetsci12050448/s1, Table S1: Survey to determine risk factors for parasitosis in backyard poultry production systems (BPPS) in Central Chile.

## References

[1] Muñoz-Gómez V., Ma T., Li Y., Rasmussen P., Torgerson P.R. (2024). Global and regional prediction of coccidiosis and ascaridiosis prevalence in extensive backyard chickens in low-income and middle-income countries. Vet. Parasitol..

[2] Laxmi N.A. (2021). Backyard poultry production and its importance. Acta Sci. Vet. Sci..

[3] Gentile N., Carrasquer F., Marco-Fuertes A., Marin C. (2024). Backyard poultry: Exploring non-intensive production systems. Poult. Sci..

[4] Correia-Gomes C., Henry M.K., Auty H.K., Gunn G.J. (2017). Exploring the role of small-scale livestock keepers for national biosecurity—The pig case. Prev. Vet. Med..

[5] Correia-Gomes C., Sparks N. (2020). Exploring the attitudes of backyard poultry keepers to health and biosecurity. Prev. Vet. Med..

[6] Abd El-Ghany W.A. (2022). An updated insight into the gastrointestinal helminthoses of poultry: A review. Ann. Parasitol..

[7] Shifaw A., Feyera T., Walkden-Brown S.W., Sharpe B., Elliott T., Ruhnke I. (2021). Global and regional prevalence of helminth infection in chickens over time: A systematic review and meta-analysis. Poult. Sci..

[8] Alcaino H., Gorman T. (1999). Parasitos de Los Animales Domesticos en Chile. Parasitol. Al Día.

[9] Mares M.M., Al-Quraishy S., Abdel-Gaber R., Murshed M. (2023). Morphological and Molecular Characterization of *Eimeria* spp. Infecting Domestic Poultry Gallus gallus in Riyadh City, Saudi Arabia. Microorganisms.

[10] Tarbiat B., Jansson D.S., Höglund J. (2015). Environmental tolerance of free-living stages of the poultry roundworm Ascaridia galli. Vet. Parasitol..

[11] Singh M., Kaur P., Singla L.D., Kashyap N., Bal M.S. (2021). Assessment of risk factors associated with prevalence of gastrointestinal parasites in poultry of central plain zone of Punjab, India. Vet World.

[12] Khursheed A., Yadav A., Sofi O.M., Kushwaha A., Yadav V., Rafiqi S.I., Godara R., Katoch R. (2022). Prevalence and molecular characterization of Eimeria species affecting backyard poultry of Jammu region, North India. Trop. Anim. Health Prod..

[13] Rafieian-Naeini H.R., Ko H., Goo D., Choppa V.S.R., Gudidoddi S.R., Katha H.R., Kim W.K. (2025). Synergistic impact of Salmonella typhimurium and *Eimeria* spp. coinfection on turkey poults: Growth performance, salmonella colonization, and ceca microbiota insights. Poult. Sci..

[14] Dohoo I.R., Martin W., Stryhn H. (2012). Methods in Epidemiologic Research.

[15] Tomazic M.L., Britez J.D., Pisón Martínez M.L., Barbano P., Canet Z., Trangoni M.D., Poklepovich T.J., Cubas F., Alegría-Morán R., Ramírez-Toloza G. (2025). Chicken Coccidiosis in Peri-Urban Family Farming in Two South American Countries: Prevalence and Circulating *Eimeria* spp.. Animals.

[16] Alegría-Morán R., Pastenes Á., Cabrera G., Fredes F., Ramírez-Toloza G. (2021). Urban public squares as potential hotspots of dog-human contact: A spatial analysis of zoonotic parasites detection in Gran Santiago, Chile. Vet. Parasitol. Reg. Stud. Rep..

[17] Soulsby E.J.L. (1984). Helminths, arthropods and protozoa of domesticated animals (7th edition): E. J. L. Soulsby, 1982. London: Baillière Tindall, 809 pp., illus. ISBN 0-7020-0820-6. Trans. R. Soc. Trop. Med. Hyg..

[18] Rinaldi L., Levecke B., Bosco A., Ianniello D., Pepe P., Charlier J., Cringoli G., Vercruysse J. (2014). Comparison of individual and pooled faecal samples in sheep for the assessment of gastrointestinal strongyle infection intensity and anthelmintic drug efficacy using McMaster and Mini-FLOTAC. Vet. Parasitol..

[19] Cheru H., Tamrat H., Hailemelekot M., Cassini R., Belayneh N. (2023). Epidemiology and identification of Eimeria species affecting poultry in East Gojjam Zone, North West Ethiopia. Vet. Med. Sci..

[20] Tiersch K.M., Daş G., von Samson-Himmelstjerna G., Gauly M. (2014). Artificial infection of chickens with Capillaria obsignata eggs embryonated in different media. Vet. Parasitol..

[21] Wickware A.B. (1947). Differential Blood Picture in Chickens: Before and after Administration of Embryonated Eggs of Heterakis Gallinae with Notes on Pathogenicity. Can. J. Comp. Med. Vet. Sci..

[22] Ikeme M.M. (1971). Observations on the pathogenicity and pathology of Ascaridia galli. Parasitology.

[23] McDougald L.R. (2020). Internal Parasites. Diseases of Poultry.

[24] Team R.C. (2023). R: A Language and Environment for Statistical Computing.

[25] Team R. (2023). RStudio: Integrated Development for R.

[26] Costa M.A., Kulldorff M., Glaz J., Pozdnyakov V., Wallenstein S. (2009). Applications of spatial scan statistics: A review. Scan Statistics. Statistics for Industry and Technology.

[27] Rao H., Shi X., Zhang X. (2017). Using the Kulldorff’s scan statistical analysis to detect spatio-temporal clusters of tuberculosis in Qinghai Province, China, 2009–2016. BMC Infect. Dis..

[28] Kulldorff M., Rand K., Williams G. (2022). SaTScan: Software for the Spatial and Space-Time Scan Statistics.

[29] Quantum G. (2018). QGIS Development Team–QGIS Geographic Information System. Open Source Geospatial Foundation Project. https://qgis.org/project/.

[30] Covacevic G., Esnaola V. (2008). Production of Eggs: Current Situation and Perspectives.

[31] Lockhart C.Y., Stevenson M.A., Rawdon T.G. (2010). A cross-sectional study of ownership of backyard poultry in two areas of Palmerston North, New Zealand. N. Z. Vet. J..

[32] Kebede A., Abebe B., Zewdie T. (2017). Study on prevalence of ectoparasites of poultry in and around Jimma town. Eur. J. Biol. Sci..

[33] Rashid M., Akbar H., Bakhsh A., Rashid M.I., Hassan M.A., Ullah R., Hussain T., Manzoor S., Yin H. (2019). Assessing the prevalence and economic significance of coccidiosis individually and in combination with concurrent infections in Pakistani commercial poultry farms. Poult. Sci..

[34] Blake D.P., Knox J., Dehaeck B., Huntington B., Rathinam T., Ravipati V., Ayoade S., Gilbert W., Adebambo A.O., Jatau I.D. (2020). Re-calculating the cost of coccidiosis in chickens. Vet. Res..

[35] López-Osorio S., Chaparro-Gutiérrez J.J., Gómez-Osorio L.M. (2020). Overview of Poultry Eimeria Life Cycle and Host-Parasite Interactions. Front. Vet. Sci..

[36] Attree E., Sanchez-Arsuaga G., Jones M., Xia D., Marugan-Hernandez V., Blake D., Tomley F. (2021). Controlling the causative agents of coccidiosis in domestic chickens; an eye on the past and considerations for the future. CABI Agric. Biosci..

[37] Belli S.I., Smith N.C., Ferguson D.J.P. (2006). The coccidian oocyst: A tough nut to crack!. Trends Parasitol..

[38] Alcaíno H., González J.P., Fredes F., Gorman T. (2002). Coccidias aviares de gallineros industriales de Chile. Parasitol. Latinoam..

[39] Torres P., Cerna O., Rubilar A., Subiabre Á., Oyarzún P. (2020). Blastocystosis y otras infecciones intestinales por parásitos eucarióticos en Gallus gallus domesticus en localidades del sur de Chile. Rev. De Investig. Vet. Del Perú.

[40] Bolfa P., Callanan J.J., Ketzis J., Marchi S., Cheng T., Huynh H., Lavinder T., Boey K., Hamilton C., Kelly P. (2019). Infections and pathology of free-roaming backyard chickens on St. Kitts, West Indies. J. Vet. Diagn. Investig..

[41] Montes-Vergara D.E., Cardona-Alvarez J., Pérez-Cordero A. (2021). Prevalence of gastrointestinal parasites in three groups of domestic poultry managed under backyard system in the Savanna subregion, Department of Sucre, Colombia. J. Adv. Vet. Anim. Res..

[42] Carrisosa M., Jin S., McCrea B.A., Macklin K.S., Dormitorio T., Hauck R. (2021). Prevalence of Select Intestinal Parasites in Alabama Backyard Poultry Flocks. Animals.

[43] Ritzi M.M., Abdelrahman W., van-Heerden K., Mohnl M., Barrett N.W., Dalloul R.A. (2016). Combination of probiotics and coccidiosis vaccine enhances protection against an Eimeria challenge. Vet. Res..

[44] Kim W.H., Chaudhari A.A., Lillehoj H.S. (2019). Involvement of T Cell Immunity in Avian Coccidiosis. Front. Immunol..

[45] Adem D.M., Ame M. (2023). Prevalence of poultry coccidiosis and its associated risk factors in and around Haramaya District, Ethiopia. Vet. Med. Open J..

[46] McDougald L.R., Cervantes H.M., Jenkins M.C., Hess M., Beckstead R. (2020). Protozoal Infections. Diseases of Poultry.

[47] Mesa-Pineda C., Navarro-Ruíz J.L., López-Osorio S., Chaparro-Gutiérrez J.J., Gómez-Osorio L.M. (2021). Chicken Coccidiosis: From the Parasite Lifecycle to Control of the Disease. Front. Vet. Sci..

[48] Kogut M.H., Lee A., Santin E. (2020). Microbiome and pathogen interaction with the immune system. Poult. Sci..

[49] Mousa M.R., Attia M.M., Salem H.M., Al-Hoshani N., Thabit H., Ibrahim M.A., Albohiri H.H., Khan S.A., El-Saadony M.T., El-Tarabily K.A. (2024). Coinfection of the gut with protozoal and metazoal parasites in broiler and laying chickens. Poult. Sci..

[50] Bautista-Vanegas A., Esteban-Mendoza M., Cala-Delgado D. (2023). Ascaridia galli: A report of erratic migration in eggs for human consumption in Bucaramanga, Colombia-case report. Arq. Bras. De Med. Veterinária E Zootec..

[51] Burrell A., Tomley F.M., Vaughan S., Marugan-Hernandez V. (2020). Life cycle stages, specific organelles and invasion mechanisms of Eimeria species. Parasitology.

[52] Tewari A.K., Maharana B.R. (2011). Control of poultry coccidiosis: Changing trends. J. Parasit. Dis..

[53] Makouloutou-Nzassi P., Longo-Pendy N.M., Nguema L.K.A., Lendzele S.S., Bangueboussa F., Bouchedi B., Maganga G.D., Boundenga L. (2024). Prevalence of gastrointestinal parasites in chickens (*Gallus gallus domesticus*) and associated risk factors in M’passa department, Southeast Gabon. Open Vet. J..

[54] Calderón E.G.Q., Colima A.B.G., Rojas Z.C. (2021). Los Factores de Riesgo Asociados a Parásitos Gastrointestinales en Animales de Producción. Cult. Científica Y Tecnológica.

[55] Alcala-Canto Y., Figueroa-Castillo J.A., Ibarra-Velarde F., Vera-Montenegro Y., Cervantes-Valencia M.E., Alberti-Navarro A. (2020). First database of the spatial distribution of Eimeria species of cattle, sheep and goats in Mexico. Parasitol. Res..

[56] Cornell K.A., Smith O.M., Crespo R., Jones M.S., Crossley M.S., Snyder W.E., Owen J.P. (2022). Prevalence Patterns for Enteric Parasites of Chickens Managed in Open Environments of the Western United States. Avian Dis..

[57] Coroian M., Fábián-Ravasz T.-Z., Dobrin P.R., Györke A. (2024). Occurrence of *Eimeria* spp. and Intestinal Helminths in Free-Range Chickens from Northwest and Central Romania. Animals.

[58] Urzúa-Encina C., Fernández-Sanhueza B., Pavez-Muñoz E., Ramírez-Toloza G., Lujan-Tomazic M., Rodríguez A.E., Alegría-Morán R. (2023). Epidemiological Characterization of Isolates of Salmonella enterica and Shiga Toxin-Producing Escherichia coli from Backyard Production System Animals in the Valparaíso and Metropolitana Regions. Animals.

[59] You M.-J. (2014). Suppression of Eimeria tenella Sporulation by Disinfectants. Korean J. Parasitol..

[60] Qin Z.R., Arakawa A., Baba E., Fukata T., Miyamoto T., Sasai K., Withanage G.S.K. (1995). Eimeria tenella Infection Induces Recrudescence of Previous Salmonella enteritidis Infection in Chickens. Poult. Sci..

[61] Madlala T., Okpeku M., Adeleke M.A. (2021). Understanding the interactions between Eimeria infection and gut microbiota, towards the control of chicken coccidiosis: A review. Parasite.

