# Supplementary material for: Risk Factors and Spatial Distribution of Gastrointestinal Parasites in Backyard Poultry Production Systems in Central Chile
Source: Vet Sci. 2025 May 7;12(5):448. doi: 10.3390/vetsci12050448 (PMC12115378; doi:10.3390/vetsci12050448)
Supplement: Supplementary file 1 [file vetsci-12-00448-s001.zip › vetsci-3580978-supplementary.pdf]

Table S1. Survey to determine risk factors for parasitosis in backyard poultry production systems (BPPS) in Central Chile.

A. BPPS identification

1. Sampling Date:
2. Name of the establishment:
3. Name of owner-operator:

B. Environmental characteristics and property access

1. Georeference:
2. Land area (m2/hectare) (can be calculated with georeference):
3. Distance from nearby farms/cities/sheds

	< 2 km	2 - 5 km	> 5 km	Not applicable
Distance between nearby sheds:				
Distance from other farms:				
Distance from another city/town:				
Public Access, Clothing, Income (Comments):				

C. Basic services

1. What is the main water source for the farm?
   a. Well
   b. Water supply network
2. What type of electrical system is used?
   a. Single-phase
   b. Triphasic
3. Is there internet access on the farm?
   a. Yes
   b. No

D. Economic and financial aspects of the operation

1. What equipment is available on the farm? (Check all that apply)
   a. Generator
   b. Solar panels
   c. Food Crusher
   d. Plucker/ defeathering machine
   e. Other:
2. Is the farm enclosed with perimeter fencing?
   a. Yes
   b. No
3. How often do you sell products through the following market channels?

	Frequently	Sometimes	Never
Local market			
Street Vending / Informal Sales			
Farmers market			
Wholesale Market Hub (Node)			
Supermarket chain			
Self-consumption			

4. Who is responsible for selling the products?

Owner-operator (male)	0	1	2	3	4	>4
Owner-operator (female)						
Adult family member (male)						
Adult family member (female)						
Underage family member						
Hired worker (male)						
Hired worker (female)						
Other:						

5. Is poultry farming the family's main source of income?
   a. Yes
   b. No
   c. Other:

E. Responsibility and management of farm personnel

1. Who works on the farm, and how many people per role?

	0	1	2	3	4	> 4
Owner-operator (male)						
Owner-operator (female)						
Adult family member (male)						
Adult family member (female)						

Underage family member						
Hired worker (male)						
Hired worker (female)						
Other:						

2. Who updates their knowledge/receives training?

Owner-operator (Male)
Owner-operator (Female)
Adult Family Member (Male)
Adult Family Member (Female)
Minor Family Member
Hired Worker (Male)
Hired Worker (Female)
No one receives training
Other:

3. Can they implement what they have learned in the training?
4. Who is responsible for the overall management of the birds?

	0	1	2	3	4	>4
Owner-operator (male)						
Owner-operator (female)						
Adult family member (male)						
Adult family member (female)						
Underage family member						
Hired worker (male)						

F. Sanitary management and animal welfare in poultry production

1. Who makes decisions about medication, disinfectants, vaccination, treatments?
   a. Contracted veterinarian
   b. State Veterinarian (INTA, PRODESAL, Municipality)
   c. Owner-operator (male)
   d. Owner-operator (female)
   e. Other:
2. Do the chickens receive any treatment? (Therapeutic drugs such as antiparasitic drugs, antibiotics)
   a. Yes
   b. No
3. What medications do you use for the birds?
4. Who is responsible for medicating the birds?

	0	1	2	3	4	>4
Owner-operator (male)						
Owner-operator (female)						
Adult family member (male)						
Adult family member (female)						
Underage family member						
Hired worker (male)						
Hired worker (female)						
Other:						

5. Who is responsible for slaughtering the birds?

	0	1	2	3	4	>4
Owner-operator (male)						
Owner-operator (female)						
Adult family member (male)						
Adult family member (female)						
Underage family member						
Hired worker (male)						
Hired worker (female)						
Other:						

G. Biosecurity and cleaning protocols in animal production

1. Disinfection of vehicles-Arches:
   a. Yes
   b. No
2. Frequency and type of cleaning
   a. By production cycle
   b. In response to specific events (e.g., excess humidity, accidentally wet bedding)
   c. Other:
3. What disinfectant do you use?

4. Who is responsible for disinfecting/cleaning the pens?

	0	1	2	3	4	>4
Owner-operator (male)						
Owner-operator (female)						
Adult family member (male)						
Adult family member (female)						
Underage family member						
Hired worker (male)						
Hired worker (female)						
Other:						

H. Environmental conditions and management systems in animal production

1. Do you have lighting system in the pens?
   a. Yes
   b. No
2. Do you have a heating system in the pen? If so, which one?
   a. Electric heating
   b. Gas heating
   c. Not applicable (No heating system)
3. Do you have a ventilation system?
   a. Yes
   b. No
4. If yes, does it include curtains?
   d. Yes
   e. No
5. If yes, what material ate the curtains made of? (Example: Plastic, fabric, mesh, etc)
6. Other ventilation methods (If any):
7. What type and quantity of drinking systems do you use?

	1-3	4-5	6-8	9-11	12-15	> 15
a. Automatic In-line						
b. Automatic Plasson-type						
c. Manual (Bucket System)						
d. Manual (Gutter System)						

8. Type of food:
9. If you use commercial feed, do you know whether it contains anticoccidials?
   a. Yes
   b. No
   c. Not sure
10. Type and quantity of feeding system do you use?

	1-3	4-5	6-8	9-11	12-15	> 15
a. Automatic						
b. Manual						

11. What elements are used for environmental enrichment?
   a. Perches
   b. Bales
   c. Enrichment Boxes
   d. Wooden Structures/ Wood Pieces
   e. Stones/Rocks
   f. Does not use enrichment
   g. Other:
12. In your opinion, does improving the birds environment result in higher profitability?
13. Size of the covered and open space:

I. Animal welfare and health monitoring

1. Do the chickens exhibit any symptoms? (Check all that apply)
   a. Diarrhea
   b. Bloody diarrhea
   c. Ruffled feathers
   d. Uneven size
   e. Respiratory symptoms
   f. Other:
2. What is the most commonly observed activity among the birds?
   a. Lying down
   b. Moving around
   c. Preening (cleaning their feathers)

d. Foraging (searching for food)
e. Eating
f. Drinking
g. Dust bathing (rolling in dirt or sand)
h. Showing apathy (lack of energy or response)
i. Isolated from the group
j. Normal behavior (according to the observer)
k. Other:

3. Do any of the birds have the following health conditions?
   a. Broken feathers
   b. Wounds
   c. Parasites
   d. Broken beaks
   e. Scaly leg mites
   f. Comb with lesions
   g. Leg wounds
   h. None
   i. Other:
4. What types of pests or infestations are present on the farm?
   a. Flies
   b. Beetles
   c. Lice
   d. Mites
   e. Other:

J. Government support

1. Is there financial compensation for mass culling due to notifiable diseases?
2. Is economic compensation provided for mass culling in cases of notifiable diseases?

K. Characteristics of the birds and production model

1. What is the type of land tenure?
   a. Private property
   b. Leased Property
   c. Public agricultural land/State-owned farmland
   d. Indigenous Community Land
   e. Other:
2. What is the type of production?
   a. Meat production
   b. Egg production
   c. Mixed (Meat & Egg)
   d. Breeding/Reproductive
3. What is the genetic line, age, and sex of the birds?
4. What is the origin of the birds, and how old are they upon arrival at the farm?
5. How many birds are currently on the farm?

L. Knowledge about coccidial disease

	Yes	No
1. Do you know about the disease?		
2. Are you aware of the impact of asymptomatic coccidiosis?		
3. Do you know its associations with other diseases?		
4. Do you consider the disease relevant?		
5. Do you implement prophylaxis?		
6. Do you conduct diagnostic evaluations?		
7. Do you carry out treatment?		

8. Have the birds been vaccinated against coccidiosis or other diseases?

M. Sample information

1. Sample Collection
   a. Time of collection
   b. External weather conditions at the time of collection
2. Sample Details
   a. Type of sample collected

b. Quantity of collected per production type and pen

N. Surveyor's observations

General management observations:

O. Surveyor's information

Full name:

ID or document number:

Workplace:
